# Multi-Replicated Enrichment Communities as a Model System in Microbial Ecology

**DOI:** 10.3389/fmicb.2021.657467

**Published:** 2021-04-09

**Authors:** Sylvie Estrela, Álvaro Sánchez, María Rebolleda-Gómez

**Affiliations:** Department of Ecology and Evolutionary Biology, Yale University, New Haven, CT, United States

**Keywords:** microbial communities, enrichment communities, mathematical models, reproducibility, community assembly, predictability

## Abstract

Recent advances in robotics and affordable genomic sequencing technologies have made it possible to establish and quantitatively track the assembly of enrichment communities in high-throughput. By conducting community assembly experiments in up to thousands of synthetic habitats, where the extrinsic sources of variation among replicates can be controlled, we can now study the reproducibility and predictability of microbial community assembly at different levels of organization, and its relationship with nutrient composition and other ecological drivers. Through a dialog with mathematical models, high-throughput enrichment communities are bringing us closer to the goal of developing a quantitative predictive theory of microbial community assembly. In this short review, we present an overview of recent research on this growing field, highlighting the connection between theory and experiments and suggesting directions for future work.

## Introduction

In the past 20 years, advances in DNA sequencing have made it possible to quantify the taxonomic and metagenomic composition of microbial communities in high throughput. This has vastly improved our understanding of the assembly process of natural and semi-natural communities, from host-associated microbiomes to host-free communities in natural and constructed environments (Human Microbiome Project Consortium, 2012; Gilbert et al., 2014; Wolfe and Dutton, 2015). The composition of these communities is often critical for the services and functions they provide and learning how to manipulate it is of great interest in fields as diverse as agriculture, medicine, and biotechnology (D’Haens and Jobin, 2019; Arif et al., 2020; Lino et al., 2020). To this end, the development of a quantitative theory that can predict shifts in community composition in response to ecological interventions is a significant aspiration.

The development of quantitative theory in any field of science requires a dialog between well-controlled experiments and mathematical modeling. The difficulties of externally controlling natural habitats, coupled with our incomplete understanding of the ecological forces at play (e.g., the precise colonization history of the human skin or the exact selective pressures in the leaf of a plant), present significant challenges for theory development. All of these challenges are circumvented in enrichment communities, which consist of natural microbiomes cultivated *ex situ* in a defined growth medium under well-controlled conditions (Hug and Co, 2018). In enrichment communities, the ecological processes that contribute to community assembly can be controlled and systematically added or removed, and their effects compared with the predictions of quantitative models that reflect the exact ecological conditions in the experiments. For instance, enrichment communities can be assembled in defined synthetic media, where the nutrient composition (at the start of each incubation) is known, as well as the temperature, the pH, osmolarity, etc. (Wagner-Döbler et al., 1998; Kucharzyk et al., 2012; Lee et al., 2013; Puentes-Téllez and Falcao Salles, 2018). Therefore, in these environments the selective pressures that shape community assembly can be in principle rationalized and incorporated into mathematical and computational models. Because the timing of inoculation events, the number of cells inoculated and the specific source of microorganisms can all be controlled too, we can also include the history of colonization and the inherent stochasticity of species arrivals into mathematical models (Marsland et al., 2019b; Chang et al., 2020b). Likewise, the frequency of nutrient additions, the dilution of cells and nutrients through the habitat, the degree to which physico-chemical conditions (temperature, pH, and light) may fluctuate over time, the connectivity between habitats, and a large number of other environmental factors can be known, and if desired, controlled externally and included in the models.

Importantly, the recent development of robot-assisted culturing platforms for large numbers of microbial communities under well-controlled conditions, both in batch and in continuous culture (Wong et al., 2018), has made it possible to conduct a large number of replicate enrichment experiments in parallel under identical conditions. The advent of low-cost, high-throughput DNA sequencing makes it possible to quantitatively characterize the composition and putative functions of these communities. This level of replication opens the way to quantitatively investigate the reproducibility of microbial community assembly and the relative strengths of stochasticity and determinism in the assembly process at different levels of organization. By quantifying the community composition across a large number of replicates over time, one may evaluate the performance of mathematical models of community assembly by their success at explaining the average abundance of species and their variability (Goldford et al., 2018; Marsland et al., 2019a).

This review aims not to provide a comprehensive overview of the use of enrichment cultures in microbial ecology throughout history. This is an old technique, dating back over a century ago to Winogradsky, Beijerinck, and other pioneers in microbiology. Other authors have recently written excellent overviews that provide newcomers with an introduction to their use (Hug and Co, 2018), and also argue, in particular, for their utility when combined with high-throughput sequencing techniques. Our goal here is narrower: we aim to highlight recent work combining high-throughput, multi-replicated enrichment communities, and mathematical modeling to elucidate the ecological principles that govern microbial community assembly. Our focus is to emphasize the ways in which the dialog between enrichment communities and mathematical modeling may help us to develop quantitative and predictive theory of microbial community assembly.

## Investigating the Reproducibility of Microbial Community Assembly

Understanding how reproducible community assembly is under different ecological conditions is essential if we wish to predict the assembly process and the response of microbial communities to perturbations. If assembly is not reproducible, then we cannot predict it. If it is reproducible, it may then become predictable as long as we learn the rules that govern it. Enrichment communities represent an ideal model system to address this question (Goldford et al., 2018; Bittleston et al., 2020; Estrela et al., 2020a,b). First, it is straightforward to eliminate (or at the very least dramatically diminish) the sources of variation among habitats that are impossible to remove in natural experiments. For instance, we can establish the large numbers of replicate synthetic habitats that contain the exact same biochemical composition, and which are exposed to the same physical factors (volume of the bioreactor, temperature, pH, etc.). We can also replicate in each of those habitats the colonization history, the frequency, size, and origin of migration events, the connectivity between the habitats, and other critical ecological processes. Since all the exogenous sources of variation across habitats can be eliminated, we are left with the endogenous (or intrinsic) sources only, such as stochastic birth and death of individuals, or stochastic species sampling from the regional species pool.

**Figure 1:**
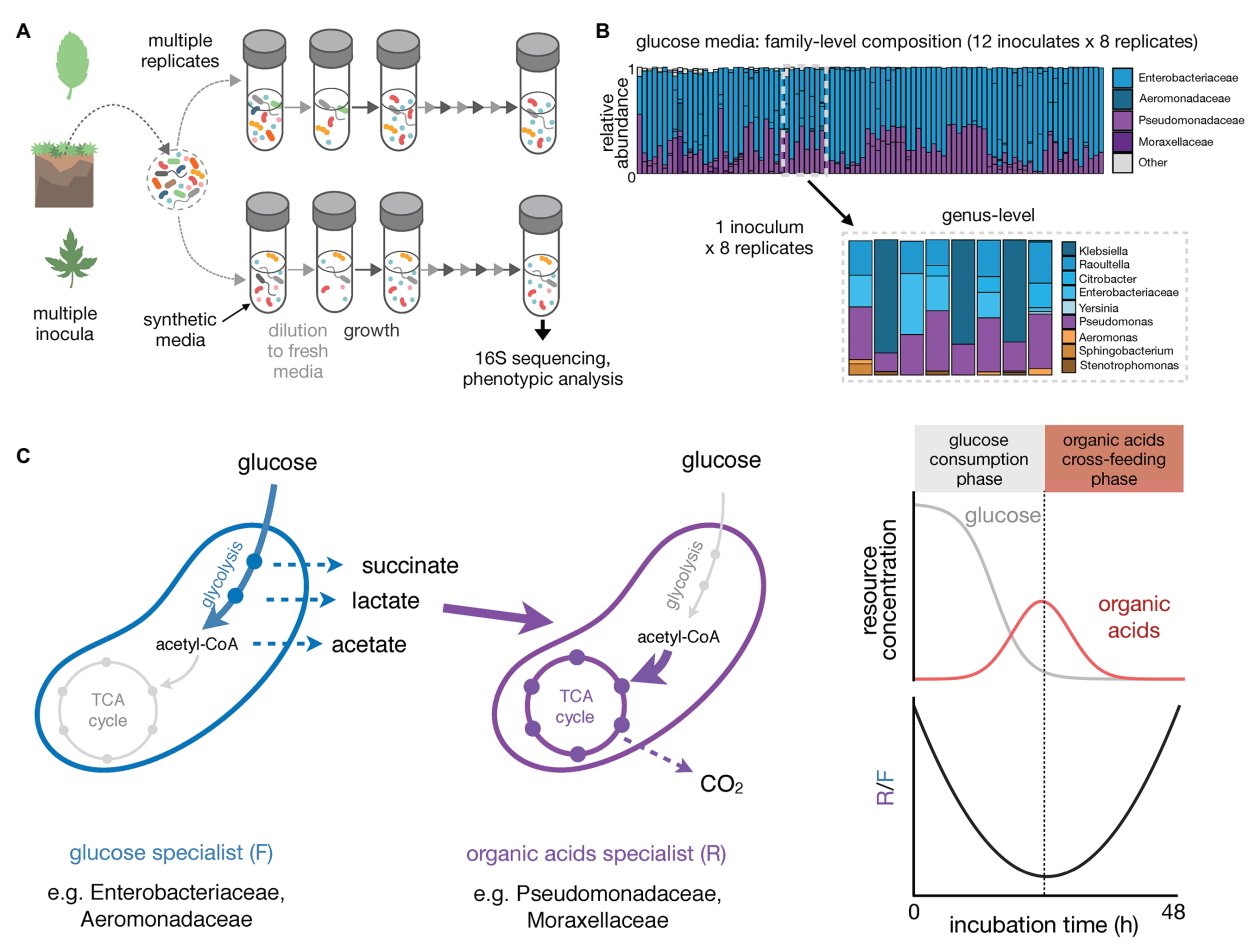
High-throughput enrichment communities to study the reproducibility of microbial community assembly. **(A)** Schematic illustrating a multi-replicated enrichment community experiment. Controlled laboratory conditions allow us to disentangle different contributions to community assembly. Natural samples are used as inocula in multi-replicate serial dilution cultures. Communities are grown in a well-defined synthetic media under well controlled conditions for a certain incubation time, followed by dilution to fresh media. This growth-dilution cycle is repeated multiple times. Community composition can be assessed using 16S rRNA amplicon sequencing. Other phenotypic assays can be performed to explore the functional properties of the self-assembled communities. **(B)** Taxonomic composition of communities self-assembled in glucose minimal media for 12 transfers of 48 h each (~84 generations in total; data from Goldford et al., 2018). The right panel shows the family-level composition of ~100 communities started from 12 different soil or plant inocula (7–8 replicates each). The left panel shows the genus-level composition of the eight replicates for one of the inocula. **(C)** Growth on minimal media with glucose as the single carbon source leads to an emergent community self-organization between two metabolic groups: glucose specialists that preferentially grow on glucose and excrete organic acids like acetate, lactate, and succinate (fermentative growth strategist, F), and organic acid specialists that preferentially grow on the organic acids (respirative growth strategists, R). The right panel shows the temporal dynamics of glucose and organic acids in the media and R/F ratio over one growth cycle (48 h-incubation). As the glucose specialists consume the glucose, the amount of organic acids in the growth media increases and the R/F ratio decreases. Once the glucose is depleted, the amount of organic acids in the media starts to decrease and the R/F ratio increases due to the growth advantage of the organic acid specialists.

To understand how reproducible microbial community assembly is, we have recently carried out experiments that monitored the assembly of hundreds of enrichment communities in a minimal synthetic medium containing a single growth limiting resource (Goldford et al., 2018). These communities were inoculated from over a dozen different environmental samples and serially passaged every 48 h for at least 80 generations (12–18 transfers; Figure 1A). In the first experiment, 12 different inocula were used, each to seed 7–8 identical replicate habitats. Communities stabilized after ~50 generations and, regardless of the identity of the single limiting nutrient (glucose, citrate, or leucine), contained N~5–25 stably coexisting species. This coexistence was largely mediated by metabolic cross-feeding, which creates additional substrates that may support N > 1 species.

One salient finding of these experiments was that repeating the same enrichment experiment multiple different times with the same inoculum often led to different community compositions at the species level, yet to strongly convergent compositions at the family and higher levels of taxonomy (Figure 1B). Family-level composition was convergent even across different inocula and was set by the specific nutrient we used. For instance, in glucose-limited minimal media all communities contained very similar fractions of the same two dominant families, Enterobacteriaceae and Pseudomonadaceae, regardless of the inoculum we used. This ratio was different in citrate, though the same two families were also dominating. In leucine, however, Enterobacteriaceae was rare and Pseudomonadaceae dominated together with other families of largely respirative bacteria (Goldford et al., 2018).

Because our enrichment communities had been assembled under well-understood and well-controlled ecological and nutritional conditions, we were able to investigate the ultimate causes for these patterns of reproducibility and divergence at different levels of taxonomy. We found that the family-level structure reflects an underlying metabolic structure (Estrela et al., 2020b; Figure 1C). In glucose media, Enterobacteriaceae are selected for their strong growth on glucose, of which they are the primary consumers. To accomplish this fast growth on glucose, Enterobacteriaceae use a respiro-fermentative metabolism, and secrete a range of overflow metabolic byproducts, such as acetate, lactate, and succinate, into the environment. These organic acids are then primarily metabolized through respiration by the Pseudomonadaceae. The conservation of quantitative metabolic traits, such as the amount of organic acids released or the growth rate in the supplied and constructed substrates, leads to the observed reproducibility at the family level (Estrela et al., 2020b). In fact, in follow-up experiments, we found that the Enterobacteriaceae can be replaced by Aeromonadaceae (a closely related family that employs a highly similar form of respiro-fermentative sugar metabolism), whereas the Pseudomonadaceae can be replaced by Alcaligenaceae (which do not even grow on glucose in M9 medium but are strong organic acid consumers).

The controlled nature of high-throughput assembly experiments make them ideal to be contrasted with theory (Dickens et al., 2016; Aguirre de Cárcer, 2019; Marsland et al., 2019a,b,c; D’Andrea et al., 2020; Worden, 2020). To this end, we have recently extended the classical MacArthur-Levins model of resource competition (Macarthur and Levins, 1967; MacArthur, 1970) to include metabolic cross-feeding, a feature that is often critical in microbial communities (Goldford et al., 2018; Marsland et al., 2019a). The main change we made to the original formulation of the model was that we allowed species to produce new resources when consuming others. We did this through an additional resource production term in the consumer-resource equations, whose most salient feature is the introduction of a stoichiometric matrix **D**, whose elements *D_ij_* capture the number of molecules of resource *i* that are secreted to the environment per molecule of resource *j* uptaken. Every species is defined in the model by a specific vector of uptake rates for the different environmental resources, including those that have been released by other taxa as well as those that were exogenously supplied.

For instance, consumer-resource models show that many of these experimental findings are generic properties of large consumer-resource systems seeded with random consumption vectors (Goldford et al., 2018). These generic properties include the fact that N > 1 species may coexist on a single supplied resource, that we observe functional convergence despite substantial variability at the taxonomic level, that multiple members of the same functional guild can stably coexist together or even the characteristic shape of the functional and population dynamics in our communities, including transient blooms of taxa (Goldford et al., 2018). By showing that these are generic properties exhibited by consumer-resource models with a large number of species, we can generalize these findings and posit that they may apply to natural communities as well, as long as their dynamics are dominated by consumer-resource interactions. Recent work has established that indeed many observed ecological patterns in natural communities are also exhibited by large communities in consumer-resource models (Marsland et al., 2019c).

In particular, the observation that communities converge functionally despite variation in taxonomy (particularly at the species level) has been made in a wide range of empirical systems, from plant associated communities to the human microbiome or the oceans (Burke et al., 2011; Human Microbiome Project Consortium, 2012; Louca et al., 2016a,b, 2018). However, recent work suggests that historical contingency may still lead to alternative functional community states. By propagating 10 different enrichment communities from pitcher plants on a nutritionally complex medium, Bittleston et al. have found that communities may not only diverge in their species composition but also in their family-level composition, as well as in many of the functions they carry out (Bittleston et al., 2020). Some emergent properties of the communities, such as their biomass or the amount of respiration, were convergent despite the taxonomic variability. Other functions, however, were fairly divergent, including those that one may reasonably assume may be important in these habitats (i.e., chitinase activity) given the growth substrate in the medium (ground insects). Another recent study by Zhou et al. reached similar conclusions. Zhou et al. examined the role of stochasticity in multi-replicated microbial electrolysis cell reactors. They found that communities assembled in identical reactors that had been colonized from the same inoculum adopted different taxonomic compositions with functionally distinct attributes (Zhou et al., 2013). Not all functions were divergent; however, for instance, methane production was fairly convergent despite substantial functional variation, whereas hydrogen production exhibited very strong differences across communities. Understanding which community-level functions are deterministically selected by the environment and under which conditions remains an important open question (Zhou and Ning, 2017). We argue here that the combination of mathematical modeling, physiological analyses, and enrichment communities is a promising approach to tackle it.

## Understanding the Factors That Control the Diversity of Microbial Communities

**Figure 2:**
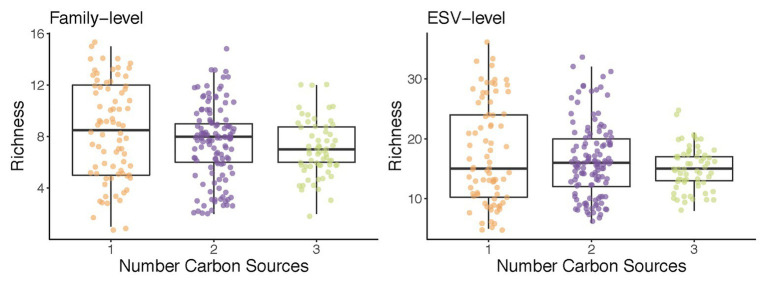
An example of the use of enrichment communities to test ecological theory: richness does not increase with the number of carbon sources supplied. Communities from two soil inocula were assembled in minimal media supplemented with one, two, or three carbon sources for 10 transfers (48 h each; Estrela et al., 2020a). Shown is the total number of families **(left)** and exact sequence variants (ESVs; **right**) in each of the self-assembled communities at transfer 10.

What drives some habitats to have a higher diversity than others? Ecological theory indicates that the number of species that can be found in a given habitat cannot exceed the number of growth-limiting resources present (Macarthur and Levins, 1967; MacArthur, 1970). It stands to reason than that, as we increase the number of supplied growth limiting nutrients, the number of taxa will increase too. Enrichment communities are an ideal system to test this hypothesis. In recent work, we have reported that increasing the number of supplied resources from 1 to 2 or 3 had a very small effect in the total biodiversity and did not lead to an appreciable increase in species richness (Estrela et al., 2020a; Figure 2). Consumer-resource models with cross-feeding recapitulated this observation, and found that the identity of the nutrients in mixed-nutrient environments (i.e., whether they were two sugars or one sugar and one organic acid) had a larger effect on biodiversity than the number of those nutrients one adds. This theoretical prediction was confirmed experimentally. In subsequent work, Bello et al. (2020) increased the number of supplied resources up to 16, and further examined the relationship between species richness and the number of (substitutable) growth-limiting nutrients. They found that, on average, a single new species was added to the community per every new nutrient in the media. This finding was also consistent with the expectations from Generalized Lotka-Volterra models parameterized according to their empirical observations, and it was also consistent with previous findings by Pacheco and Segrè (2020) using defined microbial consortia, as opposed to an enrichment approach (Pacheco and Segrè, 2020). This agreement between experimental results from different labs using different environments, starting communities, and assembly techniques points to the robustness of this rather counterintuitive finding.

Although all of these studies used carbon limited medium, one could just as easily use enrichment cultures, where other essential elements, such as nitrogen or phosphorus, are limiting (Hua et al., 2021). In addition, micro-nutrients, such as vitamins or trace elements of various minerals, can be essential for the growth of certain taxa and may be limiting biodiversity in enrichment cultures (Summers and Wyss, 1967; van Hoek and Merks, 2017; Sanchez-Gorostiaga et al., 2019). For instance, lack of vitamins may explain the conspicuous absence of *Bacillus* strains in enrichment cultures with soil bacteria in M9 medium (Goldford et al., 2018; Estrela et al., 2020a,b). Productivity in many terrestrial and aquatic communities is co-limited by multiple nutrients at the same time (Harpole et al., 2011), but the mechanisms and consequences of co-limitation are not well understood. Understanding how co-limitation affects biodiversity is a fascinating question that can be easily addressed using enrichment communities. Integrating co-factors into consumer-resource models is also possible and could lead to new insights regarding how biodiversity is generated and maintained (Dubinkina et al., 2019; Butler and O’Dwyer, 2020). Combining such theoretical advances with enrichment experiments that include an increasing number of potentially co-limiting nutrients (P, N, C, metals, vitamins, etc.) will expand our understanding of the ecological factors that govern biodiversity in microbial communities.

In addition to nutrients, other ecological factors may also play important roles in shaping biodiversity. In a recent study, Mancuso et al. (2020) examined how the rate of flow through the system affected species richness. This study differs from others in that the authors set up multi-replicated enrichment communities using an array of continuous culture devices, rather than an array of serially passaged batch cultures (Wong et al., 2018). The authors found a U-shaped dependency between species richness and flow-rate, where species richness was lowest at intermediate flow rates. The authors went on to show that when flow through the system exhibited strong fluctuations, this U-shape was lost and diversity was maintained for a wide range of dilution rates. These patterns were consistent with the behavior of mathematical models.

## Testing Ecological Hypotheses Under Well-Controlled Conditions

In the previous sections, we have highlighted and synthesized work that we and others have carried out combining mathematical models with multi-replicated enrichment communities with the aim of identifying generic quantitative patterns in microbial community assembly. Here, we propose that many other theoretical predictions and the hypotheses of ecological theory may be tested in this manner. We focus on three examples, with the hope of stimulating the reader to finding others.

For instance, in recent years there has been a surge of theoretical and empirical papers addressing the assembly of microbial communities from the bottom-up (Higgins et al., 2017; Xiao et al., 2017; Venturelli et al., 2018). An outstanding result in this field has been the finding by Friedman et al. (2017) that coexistence in communities assembled from a small number of soil isolates could be predicted by a simple assembly rule. This assembly rule states that in a multi-species mixed culture, only the subset of species that all coexist with one another in pairwise co-culture will survive, whereas those that are excluded by any member of that subset will go extinct. In small communities, this rule was found to have a strong predictive value, which declines as the communities grow in size. The authors also noted that the rule is not necessarily observed in Generalized Lotka-Volterra models. Later theoretical studies have explored in more depth the assembly paths that lead to the construction of complex communities from sequential invasion of species (Angulo et al., 2020; Serván and Allesina, 2020). These theoretical studies have posed fascinating questions, such as whether top-down and bottom-up community assembly are fundamentally different from one another and posited the existence of coexistence holes and forbidden assembly paths that, violating the empirical assembly rule described above, could be present when communities are assembled from the bottom-up. These ideas could be tested by assembling enrichment communities (which are inherently top-down), isolating their members, and then reconstituting every possible sub-community (Estrela et al., 2020b).

Another theoretically and empirically intriguing hypothesis is that diversity begets diversity in microbial community assembly (Madi et al., 2020). A mechanistic basis for this idea is that, as more species are added to a species-poor environment, more niches may be created (for instance, *via* metabolic cross-feeding), which can then recruit other taxa, paving the way for a further increase in diversity. The diversity-begets-diversity hypothesis predicts a positive relationship between the number of species within a single focal taxonomic level (e.g., genus or family), and the number of those higher taxonomic levels in the community. Analyses of a number of natural microbiomes have lent support to this hypothesis (Madi et al., 2020). Yet, interpreting it mechanistically in terms of the number of niches available and constructed is complicated in natural habitats, where microbial interactions with each other and their environment are often poorly characterized. Enrichment communities present an excellent experimental system to test this hypothesis and its mechanistic basis. First, they can be assembled in environments, where a single nutrient is supplied externally, and therefore we know for certain that the majority of niches are constructed by the microbes in the community. The number of supplied nutrients can be then increased, reducing the relative weight of niche construction. That way one could modulate the relative importance of niche construction, and assess the extent to which this reduces the correlation between, for instance, the number of species within a genus and the number of additional genera in the community. These studies could be accompanied by mathematical models that precisely reflect the conditions of these experiments, allowing us to generalize any findings and to make further predictions that could be then validated empirically.

A third example is microbial community coalescence, the process by which microbial communities invade one another (Rillig et al., 2015, 2016; Rillig and Mansour, 2017; Sierocinski et al., 2017). Recent theoretical work has laid out the conditions for which top-down assembled microbial communities would act as a cohesive unit during community coalescence (Tikhonov, 2016). Enrichment communities represent an excellent model system to contrast with theory, and find out the generic conditions under which species that coexist together will co-recruit each other when challenged by an invasive community (Sierocinski et al., 2017). We have recently showed, for instance, that the outcome of competition between the dominant species of two coalesced communities is predictive of the presence of sub-dominant taxa in the coalesced community (Lu et al., 2018). This finding is consistent with the generic behavior of consumer-resource models.

## Benchmarking Techniques Developed to Infer Properties of Natural Microbiomes

The tractability of enrichment communities allows us to start elucidating theories of microbial community assembly. In contrast, most microbial communities are very diverse and with a large number of species that we cannot (or cannot easily) grow in the laboratory (Connon and Giovannoni, 2002; Kaeberlein et al., 2002; Puspita et al., 2012). To overcome this obstacle, microbial ecologists have developed multiple computational tools to infer ecological interactions and other processes from temporal and longitudinal sequencing data. Enrichment communities allow us to not only develop new theory, but also to benchmark microbiome analysis tools under conditions where the assumptions of those techniques are known to be true.

An example is Dissimilarity-Overlap Analysis (DOA), a recently developed technique to examine the degree of variation in population dynamics and species interactions across a given sample of habitats, such as the same body site for various different hosts (Bashan et al., 2016; Kalyuzhny and Shnerb, 2017; Verbruggen et al., 2018). DOA quantifies the relationship between Dissimilarity and Overlap, two independent metrics of beta-diversity. When these two metrics are anticorrelated across a number of environments, it can be interpreted as species having similar interactions in those environments, which in turn indicates that the environments are strongly similar to one another. Yet, other alternative interpretations exist such as the possibility of an underlying environmental gradient or that only some (but not all) of those environments are very similar to one another (Kalyuzhny and Shnerb, 2017). Directly benchmarking this technique on natural samples would require a full characterization of natural environmental heterogeneity, which is exceedingly challenging (Bashan et al., 2016). To determine whether the assumptions of DOA are fulfilled under conditions, where environmental heterogeneity may be precisely controlled, we have recently applied it to hundreds of enrichment communities that include multiple replicates sharing the same (or different) inoculum and growth limiting resources (Vila et al., 2020). Our results allowed us to show that, as assumed by DOA and consistent with Generalized Lotka-Volterra simulations, when environments are identical there is a strong negative correlation between Dissimilarity and Overlap. When communities assembled in different nutrient environments are compared no correlation is seen. Yet, we also found that the presence of a negative slope between Dissimilarity and Overlap does not necessarily imply that all habitats examined are identical, and that certain taxa may be associated with deviations from this rule (Vila et al., 2020). This example illustrates that applying an analysis method to enrichment communities, where the assumptions of the method can be known to be true, helps us to understand to what extent the analysis method may be really telling us what we think it is. In other words, enrichment communities may play the role of clean negative and positive controls, whose role in the scientific method cannot be overstated.

## Final Remarks

Our hope in writing this review was to motivate the reader to think of new ways in which the combination of multi-replicated enrichment communities and mathematical models may help us to develop a quantitative understanding of how communities assemble, how different factors govern their diversity and function, and other open questions in microbial ecology. Throughout this piece, we have highlighted some of those open questions and hinted how they may be addressed. Because this piece is focused on the combination of theory and ecologically motivated studies on multi-replicated enrichment communities, we have not commented on the very large number of fascinating studies that have used enrichment communities in microbial ecology throughout its history. An entire review piece could be devoted to that topic. Just in the past couple of years, enrichment communities have been used as a means to investigate assembly mechanisms and species interactions in the gut (Aranda-Díaz et al., 2020), soil (Zegeye et al., 2019; McClure et al., 2020), and marine microbiomes (Yu et al., 2019) to generate synthetic consortia for a variety of biotechnological applications (Gilmore et al., 2019; Kang et al., 2019; Díaz-García et al., 2021) or as the substrate for artificial microbiome selection (Wright et al., 2019; Chang et al., 2020a; Sánchez et al., 2021). This list is, of course, very far from being comprehensive, and we just offer it as a small sample of the many studies that we have had to leave out for lack of space. Similarly, this short piece has focused on studies combining multi-replicated enrichment communities, high-throughput sequencing, and mathematical models. Out of this rather narrow scope are many new and creative approaches to the quantitative study of microbial communities, whether they are theoretical, (e.g., Bauer et al., 2017; Goyal et al., 2018; Dubinkina et al., 2019; Butler and O’Dwyer, 2020; D’Andrea et al., 2020), experimental, (e.g., Venturelli et al., 2018; Kehe et al., 2019), or a combination of both (e.g., Harcombe et al., 2014; Friedman et al., 2017; Dukovski et al., 2020). A number of excellent reviews have covered this growing field of synthetic ecology, with the emphasis being largely on bottom-up or synthetic approaches (Zomorrodi and Segrè, 2016; Friedman and Gore, 2017; Rodríguez Amor and Dal Bello, 2019).

Understanding the ecological dynamics and the repeatability of microbial community structure and function is important for human-health, bioremediation, biotechnology, and to understand the consequences of anthropogenic changes in ecosystem function. Unlike engineered communities from the bottom-up, microbial enrichment experiments allow us to control and manipulate the environment while avoiding making assumptions about the relevant ecological interactions and trait distributions. Thus, we can use these communities to investigate how environmental changes like increased temperatures and drought affect ecological interactions and use these results to develop predictive models of changes in metabolic structure and ecosystem function. Furthermore, and given the high overlap of ecological and evolutionary timescales for microbes, we can use multi-replicated enrichment communities to understand the effects of evolutionary change in community assembly, and how, as communities change, they affect evolution. Recent work with self-assembled communities has already shown that community context can affect the dynamics of evolution, with high diversity limiting adaptation (Scheuerl et al., 2020). The tight control over ecological and environmental factors, together with the large number of replicates that can be carried out under identical conditions, make multi-replicated enrichment communities an ideal model system to untangle the rules that govern the ecology and evolution of microbiomes.

## Author Contributions

All authors listed have made a substantial, direct and intellectual contribution to the work, and approved it for publication.

### Conflict of Interest

The authors declare that the research was conducted in the absence of any commercial or financial relationships that could be construed as a potential conflict of interest.
